# TGF-β expression and wound tensile strength after simple interrupted suturing and zip surgical skin closure (IN VIVO study)

**DOI:** 10.1016/j.amsu.2020.08.009

**Published:** 2020-08-15

**Authors:** Agus Widodo, Poerwati Soetji Rahajoe, Riyati Titi Astuti

**Affiliations:** a Resident of Department Oral and Maxillofacial Surgery, Faculty of Dentistry, Universitas Gadjah Mada, yogyakarta, Indonesia; b Staff of Department of Oral and Maxillofacial Surgery, Faculty of Dentistry, Universitas Gadjah Mada, Yogyakarta, Indonesia

**Keywords:** Zip surgical skin closure, Simple interrupted suturing, Immunohistochemistry staining, Wound tensile strength, Primary healing

## Abstract

Primary healing occurs when both edges of the adjacent incision wound meet. To achieve primary healing, bringing the wound edges closer is generally done by suturing. At present comes one of the methods of skin incision closure without involving sutures called zip surgical skin closure. As an indicator of commonly used wound healing, tensile strength is produced by collagen that involves TGF-β in its production. This study was aimed to observe the expression of TGF-β and tensile strength of the skin incision-post wound using simple interrupted suturing or zip surgical skin closure.

An experimental laboratory, this study used Sprague Dawley rats with the predetermined inclusion criteria. Thirty-six rats were applied with 3 cm-dorsal skin incisions after which they were divided into 2 groups, group 1 received simple interrupted suturing and group 2 received zip surgical skin closure. TGF-β examination was performed with BS-0086R polyclonal antibodies and wound tensile strength was observed on day 3, 7 and 14.

The independent *t*-test showed that the tensile strength of the zip surgical skin closure group was higher and was significant as observed on day 7 (p = 0.000) than that of the simple interrupted suturing group. TGF-β expression in the zip surgical skin closure group was found more numerous and significant on day 7 and 14 than that of in the simple interrupted group, (p = 0.025) and (p = 0.032) respectively. Conclusion. Skin incision-post wound healing with zip surgical skin closure is better and shows higher tensile strength and more numerous TGF-β expressions than simple interrupted suturing.

## Highlights

• Primary healing, especially healing that occurs immediately after closing edge wound.
• Tensile strength is as an indicator of commonly used wound healing produced by collagen that involves TGF-β in its production.
• The tensile strength of wound healing increases along with to the number of collagen produced and the bond between collagen matrices.
∙ TGF-β is a multifunctional growth factor, known as fibrogenic cytokines which are the key factor in stimulating the synthesis of ECM and inhibiting the process of matrix degradation

## 1 Introduction

Primary healing occurs when the two edges of the incision wound are close to one another and meet, spurs the process especially with suturing. Simple interrupted technique is prefereble because it is easy, safe, and fits the need [1,2]. At present, wound closure without suturing is increasingly popular [3]. One method of suturing-free wound closure is zip surgical skin closure as a noninvasive suture replacement material that brings wound edges closer to enable primary healing [4].

The wound healing process is a complex and overlapping process that includes the coagulation and hemostasis phase, the inflammatory phase, the proliferation phase and the remodeling phase [5]. In wound healing, Transforming growth factor-β (TGF-β) is a multifunctional growth factor, known as fibrogenic cytokines which is the key factor for stimulating the synthesis of extra cellular matrix regulators (ECM) and inhibiting the process of matrix degradation [[6], [7], [8]]. The increase in TGF-β goes along with the normal wound healing stage and will decrease if the collagen has been formed and matured [6,9].

Collagen is one of the elements that affects the tensile strength of the skin, because of which it increases according to the number of collagen produced and the bond between the collagen matrices. In addition, the tensile strength is influenced by the shape of the collagen web, collagen fiber bundle density and its chemical composition [[10], [11], [12]]. The skin tensile strength is objectively the method used for evaluating wound healing and is commonly used in experimental studies [13].

The absence of puncture wounds and thread irritation which adds to inflammation in the use of zip surgical skin closure is expected to stimulate more TGF-β expression and higher tensile strength. This study was aimed at comparing the wound healing of skin incision in the use of simple interrupted suturing compared to zip surgical skin closure, as seen from TGF-β expression and incision-post skin tensile strength.

## 2 Materials and methods

This experimental laboratory study was approved by the research ethics commission of the Faculty of Veterinary Medicine, Gadjah Mada University (0012/EC-FKH/Eks/2019). Subjects consist of 36 Sprague Dawley rats with inclusion criteria such as male, aged 3–4 months, ± 200–300 g in weight, healthy, controlled post-surgical bleeding, no infection, stable weight and exclusion criteria such as uncontrolled bleeding, infected and dead rats.

### 2.1 Treatment group

In all subjects incision wounds were made in the dorsal cranial to caudal direction and using a simple random method, the subjects were split into 2 groups, each of which consists of 18 rats., The rats in group I were sutured by simple interrupted technique and those of group II received zip surgical skin closure. On day 3, 7 and 14, 6 rats of each group were decapitated to measure tensile strength and to observe TGF-β expression. During the period of study, weighing and clinical observation of wound were performed with Southampton criteria [14].

### 2.2 Incision wound preparation

The incision was performed on the back of the rat with a distance of 1.5 cm from the midline, 3-cm long with a deep subcutaneous cranial to caudal direction. Incision was done under general anesthesia with injection of 10% ketamine (100 mg/kg BW) (0.3 ml) and xylazine (10 mg/kg BW) (0.15 ml) intra peritoneal. Hair was shaved and sterilized using 10% alcohol and iodine solution. Incision wound was made with the use of scalpel no. 15 (Lotus®, China).

**Fig. 1:**
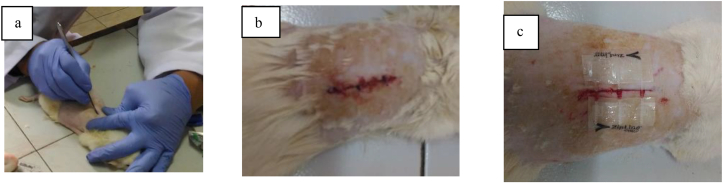
(a) Incision wound is made on a mouse's back (b) Incision wound is done with simple interrupted sutures (B Braun® Spain) (c) Incision wound is closed with zip surgical skin closure (Zipline® Medical, USA).

Group I (simple interrupted) incision wound was made closer using 3 simple interupted knot sutures with nylon 4.0 (B Braun® Spain) and group II (zip surgical skin closure) was made closer using a 4-cm long zip surgical skin closure (Zipline® Medical, USA). After the procedure, all were given intramuscularly gentamicin sulfate 2–4 mg/kg BW/24 h and paracetamol 10 mg/kg/8 h orally for 3 days. Observation was done to know the rats’ general condition, clinical condition of wound with Southampton criteria [14] and body weighing (see Fig. 1).

### 2.3 Sampling

**Fig. 2:**
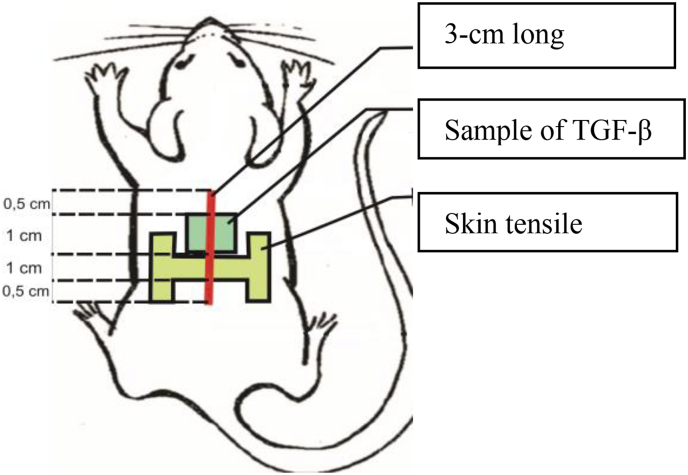
Pattern and size of sampling for TGF-β expression and skin tensile strength.

6 rats were decapitated in each group on day 3, 7, and 14 with general anesthetics by mixing 10% ketamine (100 mg/kg BW) 0.3 ml and 2% xylazine (10 mg/kg BW) intraperitoneal, after which cervical dislocation was performed. Samples for observing the tensile strength and expression of TGF-β were taken from the incision wound made. For samples of tensile strength, it was 0.5 cm from caudals and 1 cm in width and for TGF-β expression sample, it was 1 cm wide with a distance of 0.5 cm from cranial as seen in Fig. 2.

### 2.4 Measurement of tensile strength

Skin tensile strength was measured with Tensile strength tester (Pearson®, UK) by which skin was pinched at both ends according to a pattern made then the machine was activated to cut the incision line and the value on the monitor was recorded and divided by the cross-sectional area of the skin (N/cm2).

### 2.5 Assesement of TGF-β expression

Samples that have been taken were put in 10% formalin solution, after which they were included into a process of fixation, dehydration, clearing, paraffin infiltration, embedding and cutting in 4-μm thick in the transverse direction parallel to the transverse axis with the microtome (Leica®, Germany). Immunohistochemical staining used TGF-β BS-0086R polyclonal antibodies (Bioss®, USA) according to the factory's staining procedure. Observation of the amount of TGF-β expression was carried out with a light microscope (Olympus® cx 23, Japan) with a magnification of 100x to see all fields of view by an anatomic pathologist (EM), after which it was increased by 400× magnification and divided into 6 random fields. TGF-β expression calculation was performed with ImageJ software (National Institute of Health, Bethesda, USA) and matrix laboratory (Matlab) (Mathworks, USA).

### 2.6 Statistical analysis

Data obtained from observing of tensile strength and TGF-β expression were processed with IBM SPSS version 23.0 (IBM Corp., Armonk, USA) statistical application. Data normality test was done by Shapiro-Wilk and homogeneity test with Levene's test continued with Anova test and independent *t*-test. The Spearman test was performed to determine the correlation between TGF-β expression and tensile strength in each observation group.

## 3 Results

The evaluation of the rats' general condition during the research revealed that the rats were healthy, none died, good in mobility, no rats experienced an infection, and the rat's body weight increased (Table 1).

**Table 1:** Rat's weight during the research period.

Group (n)	Day 0 (18) $\bar{\text{x}}\pm SD$	Day 3 (18) $\bar{\text{x}}\pm SD$	Day 7 (12) $\bar{\text{x}}\pm SD$	Day 14 (6) $\bar{\text{x}}\pm SD$	p
I	260.5 ± 19.8	260.9 ± 20.1	278.9 ± 12.9	293.1 ± 14.4	0.013*
II	273.2 ± 34.6	273.4 ± 34.6	274.9 ± 20.1	293.3 ± 12.6	0.038*

$\bar{\text{x}}$: mean Group I: simple interrupted suturing.

SD: Standard Deviation Group II: zip surgical skin closure.

P: ANOVA test (α = 95%).

*: significantly different.

**Fig. 3:**
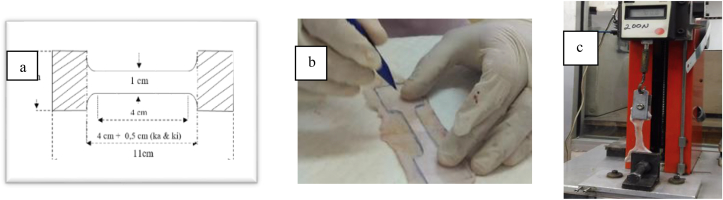
(a) Pattern of cutting skin for tensile strength test (b) Cutting tensile strength test sample (c) Tensile strength measurement with tensile strength tester (Pearson®, UK).

**Fig. 4:**
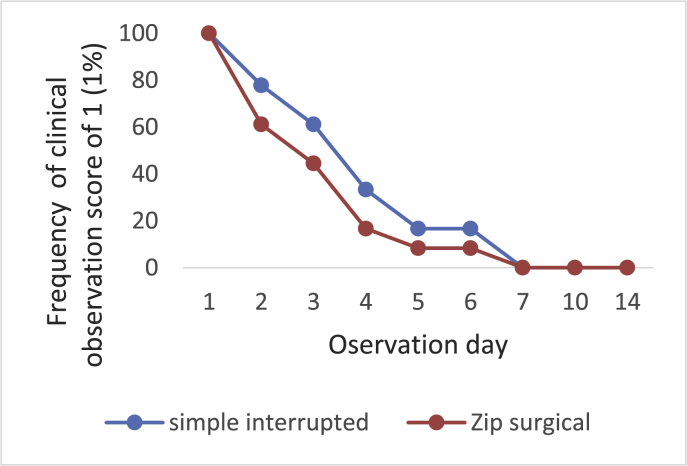
Clinical wounds with a score of 1 (Southampton index) during the research.

The clinical condition of skin incision wound was observed with the Southampton index [14] with a score of 0–5 (see Fig. 3). Score 0: normal healing, score 1: normal healing with slight bruises and erythema, score 2: normal healing with numerous erythemas and signs of inflammation, score 3: clear or reddish fluid is present, score 4: pus in the wound occurs, score 5: deep and severe skin infection with or without tissue damage. During the study the condition of clinical wound was in score 1 and during observation day 7 and 14 all wounds experienced normal healing (score 0) (Table 2). In all groups the number of score 1 seemingly decreased over time, but group II (zip surgical skin closure) showed smaller number of score 1. This result indicates that the clinical healing of the zip surgical skin closure group was better than that of simple interrupted suturing (Fig. 4)

**Table 2:** Percentage of clinical wounds with a score of 1 (Southampton index).

Group	Observation Day (n)							
	1 (18)	2 (18)	3 (18)	4 (12)	5 (12)	6 (12)	7(12)	14(6)
I n(%)	18(100)	4(77.78)	11(61.11)	4(33.3)	2(16.67)	2(16.67)	0(0)	0(0)
II n(%)	18(100)	11(61.11)	8(44.44)	2(16.67)	1 (8.33)	1 (8.33)	0(0)	0(0)

Group I: simple interrupted suturing.

Group II: zip surgical skin closure.

### 3.1 Skin tensile strength

**Fig. 5:**
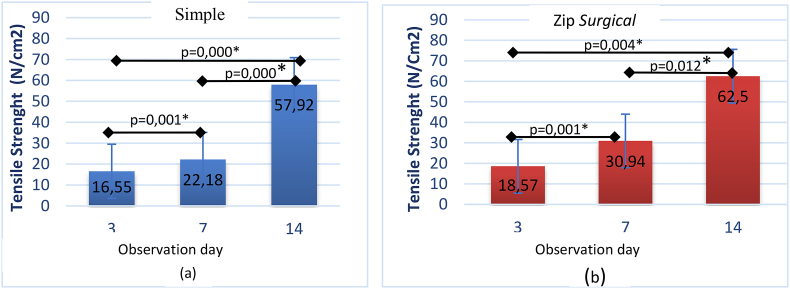
Differences in skin tensile strength of the simple interrupted suturing group (a) zip surgical skin closure (b) between times in each group.

The results of Shapiro-Wilk normality test and Levene's homogeneity test of post-incisional wound tensile strength test in each group showed normal and homogeneous distribution. Anova test (α = 95%) of the skin tensile strength of each group on 3 observation days indicated a significant difference. Independent *t*-test in the simple interrupted suturing group among observation days showed significant results, the same value was found in the group of zip surgical skin closure (see Fig. 5).

**Fig. 6:**
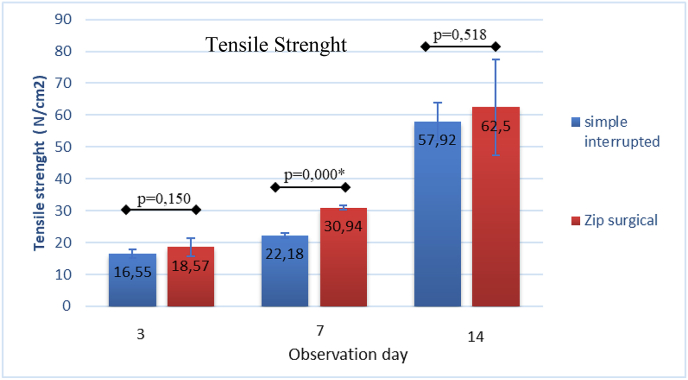
Difference in mean of skin tensile strength between groups based on observation days.

**Fig. 7:**
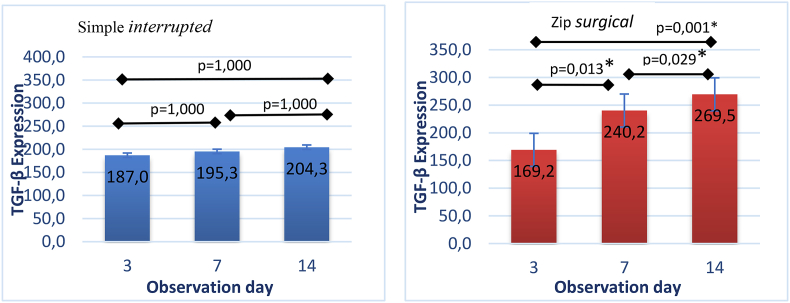
a. Differences in TGF-β expression in group of the simple interrupted suturing (a) and zip surgical skin closure (b) between observation days.

The result of independent *t*-test of the zip surgical skin closure group on day 7 was significantly greater than that of the simple interrupted suturing group (p = 0.000), whereas day 3 and day 14 were likely greater despite significance with p = 0.150 and p = 0.518 (Fig. 6). This generally shows that the zip surgical skin closure group has better skin tensile strength than simple interrupted suturing (see Fig. 7).

### 3.2 TGF-β expression after skin incision

The results of Shapiro-Wilk normality test and Levene homogeneity test showed TGF-β expression data of post-incisional skin wound were normally distributed and homogeneous. Anova TGF-β test results of the simple interrupted suturing group showed no significant results on the whole observation days (p = 1.000), whereas in the zip surgical skin closure group, significant results were found as showed in the value of the independent *t*-test on day 3 and day 7 (p = 0.013), day 3 and day 14 (p = 0.001), day 7 and day 14 (p = 0.029)

**Fig. 8:**
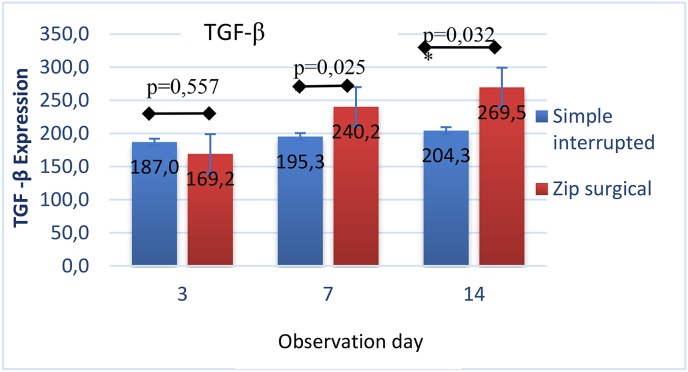
Differences in mean of TGF-β expression between groups in each observation.

**Fig. 9:**
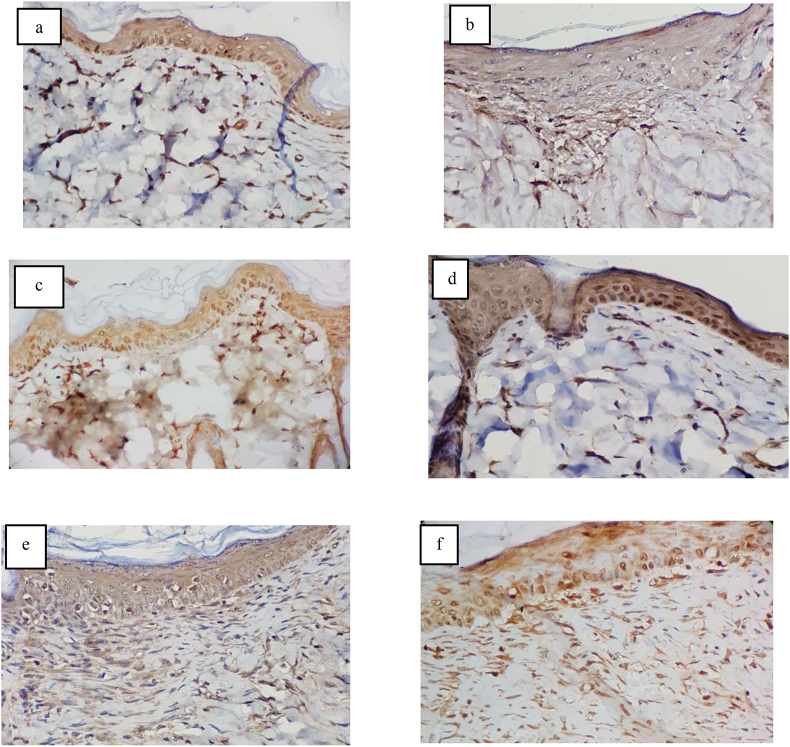
Staining of TGF-β expression with IHC BS-0086R (Bioss®, USA) (a). Day 3 of the simple interrupted suturing group (b). Day 3 of zip group surgical skin closure (c). Day 7 of Simple interrupted suturing group (d). Day 7 of Zip expressions of surgical skin closure (e). Day 14 of the simple interrupted suturing (f) group. Day 14 of the zip surgical skin closure group.

Differences in TGF-β expression between the two groups by independent *t*-test based on observation days (Fig. 8), showed TGF-β expression on day 7 and day 14 of the zip surgical skin closure group was more significant with (p = 0.025) and (p = 0.032) respectively. Different results, however, occurred on day 3, the zip surgical skin closure group showed no significant difference (p = 0.557) with a lower tendency. In general the results consistently show that the zip surgical skin closure group is better than simple interrupted suturing in wound healing of skin incision (see Fig. 9).

### 3.3 Correlation between TGF-β expression and skin tensile strength after skin incision

**Fig. 10:**
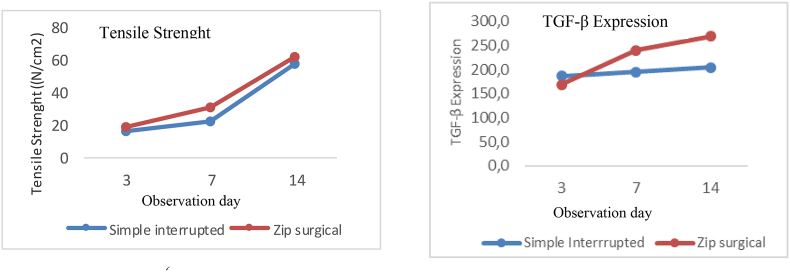
(a) Pattern of skin tensile strength between simple interrupted suturing and zip surgical skin closure (b) TGF-β expression patterns between simple interrupted suturing and zip surgical skin closure group.

The pattern of tensile strength of the wound between simple interrupted suturing and zip surgical skin closure was likely to be identical, which increased in both groups until the end of observation, but the tensile strength in the zip surgical skin closure group was found higher (Fig. 10a). Different results were seen in graphic patterns of TGF-β expression because the zip surgical skin closure group on day 3 was lower, but higher after day 7 and 14 depsite similar pattern (Fig. 10b).

The Spearman correlation test between tensile strength and TGF-β in both groups showed a significant correlation (Table 5). The zip surgical skin closure group showed stronger correlation (r = 0.713) than the simple interrupted suturing group, simply indicating that the zip surgical skin closure is better than simple interrupted suturing.

**Table 5:** Correlation between tensile strength and TGF-β in group of simple interrupted suturing and zip surgical skin closure.

Group	r	p
Simple interrupted suturing	0.532	0.023 *
Zip surgical skin closure	0.713	0.001*

r: Correlation coefficient.

p: Spearman's correlation test.

*significant correlation (α = 95%).

## 4 Discussion

Skin tensile strength refers to the maximum level of force required to pull or tear the skin to break up divided by the wound size.[14,15] Some studies used skin tensile strength as an indicator of wound healing especially in experimental studies with the use of tensile strength tester.[13,[16], [17], [18]] The tensile strength of skin wounds is produced by the number and bond among collagen which is indirectly affected by cytokine activity, one of which is TGF-β which increases the collagen matrix and is locally influenced by the size and shape of the wound, hematoma in the process of healing, infection, mechanical stress, dressing, wound covering material, suturing technique, antibiotic use, tissue type and wound care[13,[19], [20], [21]]

The research model used in this study is a wound with primary healing, especially healing that occurs immediately after closing edge wound [22]. The tensile strength in the zip surgical skin closure group showed better result than the simple interrupted suturing group as found in all groups. A higher tendency occurred in all groups although significant differences were only observed on day 7 (p = 0.000) (Fig. 6). This condition was consistent with the observation of clinical wound that occurred, seen in the zip surgical skin closure percentage at score 1 (Southamton index) showed lower level than the simple interrupted suturing group, suggesting better output of the zip surgical skin closure group in wound healing (Table 2). However, the short-sized incision wound located in the dorsal which is stable became the weakness of this study.

The tensile strength of wound healing increases along with to the number of collagen produced and the bond between collagen matrices. The tensile strength is also affected by the shape of the collagen web, the collagen fiber density, and its chemical composition.[12, 15,16]. Once wound healing takes place, collagen deposition will begin to occur and the wound tensile strength will also increase [13]. Mustika's study in rats proved that the collagen density was higher in the use of plasters compared to simple interrupted sutures especially the one without sutures to resemble zip surgical skin closure [22]. This study found that the tensile strength of the zip surgical skin closure group was also higher (p = 0.000) compared to the simple interrupted suturing group.

In addition to collagen, inflammation will also affect the tensile strength of skin wound. As found in this study the presence of puncture wound and thread irritation will reduce tensile strength. The simple interrupted suturing group showed greater inflammation (Southampton index) but lower tensile strength than zip surgical skin closure (p = 0.000). Gurtner's study found that wound closure without sutures will provide better tissue perfusion [23]. Skin wound closure by suturing will increase inflammation that spurs more macrophages around the wound to affect the wound healing process [24].

Transforming growth factor-β (TGF-β) is a multifunctional growth factor, known as fibrogenic cytokines which are the key factor in stimulating the synthesis of extracellular matrix regulators (ECM) and inhibiting the process of matrix degradation [6]. The role of TGF-β at each stage of wound healing is very important as growth factors that are molecularly and cellularly interrelated. TGF-β is produced by platelets, macrophages, fibroblasts, keratinocytes and endothelial cells, which contribute considerably in the healing process of acute and chronic wounds [8]. Day 3 observation found that TGF-β expression in the zip surgical skin closure group was lower compared to the group simple interrupted suturing despite insignificance, while higher and more significant results were observed on day 7 and 14 (p = 0.025 and 0.032) leading to faster wound healing in the zip surgical skin closure group.

Observation on day 3 showed that the value of TGF-β expression of the simple interrupted suturing group was higher, with lower and insignificant tensile strength (p = 0.557). However, day 14 showed insignificant tensile strength in the zip surgical skin closure group compared the simple interrupted group but TGF-β expression was found significantly higher (p = 0.032). On day 3, cells formed were dominated by inflammatory cells influenced by TGF-β, so that collagen-forming cells were rare [17]. Despite not observing inflammatory cells but as Koh and Dipietro said that on day 3 wound healing was dominated by inflammatory cells.[25,26] and TGF-β also increased along side the increase in inflammatory cells.

The role of TGF-β in the inflammatory stage is to attract macrophages and neutrophils, reduces the inflammation that occurs and regulates cell immunity and expresses TGF-β, so the formation of ECM and the maturation of collagen end up in less optimal function.[27,28]. The presence of greater inflammation causes tensile strength in lower interrupted suturing simple groups. Healing process on day 14 on both groups came to the final stage of proliferation and clinically the wound closed completely, as evident in the Southampton index, on which both groups reached a score of 0. In theory this period marks the beginning of collagen degradation, wound contraction, compaction of connective tissue, and epithelial formation, influenced by keratinocyte cells that express TGF-β, Therefore, TGF-β expression remains significant in value which contributes epithelialization of wound tissue, not as a cytokine.[[28], [29], [30]] It is also evident in this study that there was a positive correlation between the tensile strength of skin wound and TGF-β.

## Conclusion

Skin tensile strength and TGF-β expression were found better in the use of zip surgical skin closure compared to that of simple interrupted suturing. The tensile strength on observation day 7 and 14 (p = 0.025 and 0.032) respectively and TGF- expression β was significant on observation day 7 (p = 0.000).

## Funding

This research was self-funded, there was no sponsor in this study.

## Author contribution

Poerwati Soetji Rahajoe DDS: study design, concept, data analysis, data interpretation, writing the paper. Agus Widodo DDS: study design, data collection, data analysis, writing the paper. Riyati Titi Astuti DDS: study design, data interpretation.

## Registration of research studies

1.Name of the registry.

2.Unique Identifying number or registration ID.

3.Hyperlink to your specific registration (must be publicly accessible and will be checked).

## Guarantor

Poerwati Soetji Rahajoe, DDS.

Agus Widodo, DDS.

## Ethical approval

This research has had ethical approval from Faculty Vetenary Medicine, Universitas Gadjah Mada, Yogyakarta (0012 / EC-FKH / Eks / 2019).

## Provenance and peer review

Not commissioned, externally peer reviewed.

## Declaration of competing interest

The authors declare that they have no conflicts of interests.
